# Special Issue “Frontiers in Nucleic Acid Chemistry—In Memory of Professor Enrique Pedroso for His Outstanding Contributions to Nucleic Acid Chemistry”

**DOI:** 10.3390/molecules28217278

**Published:** 2023-10-26

**Authors:** Montserrat Terrazas, Ramon Eritja, Daniela Montesarchio

**Affiliations:** 1 Department of Inorganic and Organic Chemistry, Organic Chemistry Section, Institute of Biomedicine of the University of Barcelona (IBUB), University of Barcelona, 08028 Barcelona, Spain; 2 Institute for Advanced Chemistry of Catalonia (IQAC), CSIC, 08034 Barcelona, Spain; 3 Centro de Investigación Biomédica en Red de Bioingeniería, Biomateriales y Nanomedicina (CIBER-BBN), Instituto de Salud Carlos III, 28029 Madrid, Spain; 4 Department of Chemical Sciences, University of Naples Federico II, I-80126 Napoli, Italy

This Special issue is dedicated to the memory of Enrique Pedroso, Professor Emeritus of Organic Chemistry at University of Barcelona, who passed away at the age of 72 in September 2020. Professor Enrique Pedroso has been one of the pioneers of Nucleic Acids Chemistry in Spain, significantly contributing to the development of this highly interdisciplinary field which combines organic chemistry, biochemistry, pharmacology, materials chemistry, and biophysics. His major research achievements have been accomplished in the synthesis of modified oligonucleotides and especially conjugates and cyclic oligonucleotides, as well as their analogues, which opened new avenues in the search for novel applications of oligonucleotides.

His research activity started in 1981 at the Department of Organic Chemistry of University of Barcelona, where he spent all his intense academic and scientific career, covering almost four decades. After having deeply investigated the methods of solid phase peptide synthesis, providing significant progress in the field also in collaboration with Ernest Giralt, Fernando Albericio and Ramon Eritja of the same University, in 1990, he published his first article on the solid-phase synthesis of oligonucleotides [1]. This work was immediately followed by an important contribution on the synthesis and characterization of oligodeoxynucleotides containing the mutagenic base analogue 4-*O*-ethylthymine, carried out in collaboration with Ramon Eritja [2], with whom he always maintained strict research relationships.

After an enlightening research stay in University of Colorado in Boulder, hosted in the group of prof. Marvin H. Caruthers, he grew a solid and active research group starting from the early 1990s in collaboration with Anna Grandas—his closest collaborator in research and beloved partner in life. Together, they developed efficient approaches for the synthesis of hybrid compounds, such as nucleopeptides and peptide–oligonucleotide conjugates, among others (see: [3,4,5,6]).

In 1997, he published an effective method for the solid-phase synthesis of cyclic oligodeoxyribonucleotides, successfully engineering the functionalized solid support so to exploit phosphotriester chemistry for the crucial cyclization step of the target oligonucleotide, previously assembled via standard phosphoramidite chemistry protocols [7]. Later, this approach was extended to the synthesis of cyclic oligoribonucleotides [8]. Using this methodology, his group synthesized several small and medium-sized cyclic oligonucleotides, studying their peculiar properties and discovering unusual conformational motifs, also in collaboration with the research group of Carlos Gonzalez [9,10,11]. A more recent contribution revisited the thiol–maleimide condensation reaction to obtain cyclic oligonucleotide constructs [12].

If most of his scientific collaborations were with eminent Spanish researchers, relevant was also his international profile, being an invited speaker in many international conferences, Visiting Professor in many Universities outside Spain and having a number of fruitful international collaborations, e.g., with Eric T. Kool [13], Daniela Montesarchio [14] and Keith Fox [15], among others. In his last contribution, always collaborating with Anna Grandas, he explored the synthesis of oligonucleotide, peptide or PNA conjugates, successfully obtained via inverse electron-demand Diels−Alder cycloaddition [16].

In all his career, Enrique was deeply involved in the research and promotion of Nucleic Acid Chemistry. He was indeed an active member of the International Society for Nucleosides, Nucleotides and Nucleic Acids (IS3NA, www.is3na.org), which organizes International Roundtables on a biannual basis to present and discuss advances in chemistry and biology of nucleosides, nucleotides and nucleic acids, as well as of the Spanish Society for Nucleic Acids and Nucleosides, which gathers researchers every two years in the Spanish Nucleosides Nucleotides and Nucleic Acids meetings (RANN).

This Special issue comprises a collection of twelve research or review articles prepared by international experts in nucleic acids, including nucleoside and oligonucleotide synthesis, nucleic acids structural studies, DNA repair, and biophysical characterization of DNA-targeting ligands, especially G-quadruplex binding drugs.

A novel route for the synthesis of 2′,3′-dideoxy and 2′,3′-didehydro nucleosides, including several anti-HIV drugs such as stavudine, zalcitabine and didanosine, was presented by the group of Dr. Ferrero and Dr. Fernández (contribution 1) from the University of Oviedo. Starting from the protection at the 5′-hydroxyl group of the corresponding ribonucleotides followed by the formation of the corresponding 2′,3′-bisxanthates, a key step in the synthesis of the target compounds was the radical deoxygenation of the bisxanthates, successfully realized using environmentally friendly and low-cost reagents.

Phosphorodiamidate morpholino oligomer (PMO) derivatives are extensively used in exon-skipping strategies for the treatment of Duchenne muscular dystrophy, but the preparation of these derivatives is not trivial at all. Prof. Caruthers provided a comprehensive review on the synthesis and properties of modified morpholino oligonucleotides developed by his group at the University of Colorado in Boulder (contribution 2). This work is a masterpiece in the field of nucleic acid chemistry showing the state-of-the art of phosphoramidite chemistry, nucleoside chemistry and development of novel protecting groups for oligonucleotide synthesis.

An exceptional development in siRNA therapeutics is the discovery and use of oligonucleotide conjugates carrying trivalent N-acetyl galactosamine (GalNAc) residues. These oligonucleotides are rapidly internalized via the clathrin-mediated pathway in hepatocytes due to the presence of an asialoglycoprotein receptor with high affinity for galactose glycoproteins and trivalent GalNAc oligonucleotides. Eritja et al. at the IQAC-CSIC in Barcelona demonstrated that the tetramerization of G-rich oligonucleotides may be a novel and simple route to obtain the beneficial effects of multivalent N-acetylgalactosamine functionalization (contribution 3,4).

Meschaninova et al. from Novosibirsk State University described novel methods for the 5′-functionalization of oligonucleotides through acid labile phosphoramidate linkages (contribution 5). A wide variety of oligonucleotides 5′-conjugated with ligands, such as cholesterol, oleylamine, and p-anisic acid, was described. The methodology was successfully applied to DNA, RNA, and 2′-O-methyl-RNA oligonucleotides.

The possibility of using enzymes for the preparation of modified oligonucleotides was addressed by Hollenstein et al. from the University of Paris (contribution 6). In this work, the enzymatic synthesis of a modified nucleoside triphosphate equipped with a vancomycin moiety on the nucleobase was described, demonstrating that this nucleotide analogue is suitable for polymerase-mediated synthesis of modified DNA and compatible with the SELEX methodology for the production of aptamers suitable to fight bacterial resistance.

The impact of the duplex-G-quadruplex equilibrium in DNA Base Excision Repair was studied by Sowers et al. from the University of Texas Medical Branch (contribution 7), suggesting that DNA damage and repair intermediates can alter duplex-quadruplex equilibrium. To corroborate this hypothesis, the authors used G-quadruplex stabilizing compounds, such as pyridostatin, modified oligonucleotides containing uracil, 5-hydroxymethyluracil, 5-fluorouracil, as well as abasic sites as building blocks inserted into the loop region of a 22-base telomeric repeat sequence known to form stable and well-characterized G-quadruplex structure.

In addition to G-quadruplex structures formed by G-tetrads, other tetrads can be formed. These structures found in some constrained oligonucleotides are more frequent than expected. An excellent review by Gonzalez et al. summarized the present state-of-the-art on our knowledge on novel non-G-tetrads including homotetrads, as well as major and minor groove tetrads, emphasizing their peculiar structural features (contribution 8).

Strand-invading approaches using chemically modified oligonucleotides and nucleic acid mimics capable of unzipping Watson–Crick base pairs of dsDNA targets and forming new Watson–Crick base pairs between probe strands and the complementary DNA (cDNA) regions have been explored by Hrdlicka et al. from the University of Idaho (contribution 9). The use of densely modified oligonucleotides with 2′-O-(pyren-1-yl)methyl RNA pyrimidine building blocks is highly recommended to achieve satisfactory results.

Aptamers are nucleic acid molecules able to selectively recognize several substrates, including small molecules and proteins, acting in a similar way as antibodies. Liu et al. analyzed the intrinsic fluorescent properties of DNA for the characterization of the binding of several model aptamers to their targets, such as cortisol, Hg^2+^, adenosine or caffeine (contribution 10). They found that some aptamers may induce changes in intrinsic fluorescence, but these changes cannot be used for the determination of binding constants.

G-quadruplexes are important non-canonical DNA structures that are present in G-rich regions such as telomeres and some promoter regions of oncogenes. The polymorphism of these structures and the possibility of stabilizing them using planar heterocyclic small molecules is one of the most intense areas of research in nucleic acid chemistry. Dallavalle et al. from the University of Milan explored the interaction of several G-quadruplex ligands such as Curaxin, CX 5461, BA41, TPHS 4, Pyridostatin or BMH 21 with a G-quadruplex sequence found in the PARP1 promoter region (contribution 11). Pyridostatin was found to be the best binder for this sequence, being able to adapt its planar but flexible conformation to the dynamic nature of the G-quadruplex, especially the hybrid 3 + 1 G-quadruplex structure found in the PARP 1 promoter region.

Oliviero et al. studied the interaction of the c-myc oncogene NHE III1 region with the 3-β-D-glucoside of trans-resveratrol (Polydatin) (contribution 12). The experimental and modelling data show that this compound may be involved in partial end-stacking to the terminal G-quartet. Moreover, H-bonding interactions between the sugar moiety of the ligand and deoxynucleotides not included in the G-tetrads are possible.

Expansion of short nucleotide repeats is one of the causes of genetic diseases and is dramatically crucial for neurological diseases such as amyotrophic lateral sclerosis and frontal temporal dementia. Wang et al. described the state-of-the art knowledge of the structural properties of GGGGCC repeats in RNA and their interaction with small molecules and proteins specifically binding to these repeats (contribution 13).

By collecting in these scientific works this Special Issue, prepared by international leaders in the fields of chemistry and biochemistry of nucleosides, nucleotides and nucleic acids, we hope to contribute to advance knowledge on these fascinating molecules and their almost infinite applications, following Enrique Pedroso’s lifelong commitment and example.
